# Expression of myogenes in *longissimus dorsi* muscle during prenatal development in commercial and local Piau pigs

**DOI:** 10.1590/1678-4685-GMB-2015-0295

**Published:** 2016-10-31

**Authors:** Evelyze Pinheiro dos Reis, Débora Martins Paixão, Otávio José Bernardes Brustolini, Fabyano Fonseca e Silva, Walmir Silva, Flávio Marcos Gomes de Araújo, Anna Christina de Matos Salim, Guilherme Oliveira, Simone Eliza Facioni Guimarães

**Affiliations:** 1 Departamento de Zootecnia, Universidade Federal de Viçosa (UFV), Viçosa, MG, Brazil; 2 Departamento de Bioquímica Agrícola, Universidade Federal de Viçosa (UFV), Viçosa, MG, Brazil; 3 Fiocruz, Centro de Pesquisas René Rachou, Belo Horizonte, MG, Brazil; 4 Vale Technology Institute, Belém, PA, Brazil

**Keywords:** commercial line, gene expression, myogenesis, pig, Piau breed

## Abstract

This study used qRT-PCR to examine variation in the expression of 13 myogenes during muscle development in four prenatal periods (21, 40, 70 and 90 days post-insemination) in commercial (the three-way Duroc, Landrace and Large-White cross) and local Piau pig breeds that differ in muscle mass. There was no variation in the expression of the *CHD8, EID2B, HIF1AN, IKBKB, RSPO3, SOX7* and *SUFU* genes at the various prenatal ages or between breeds. The *MAP2K1* and *RBM24* genes showed similar expression between commercial and Piau pigs but greater expression (p < 0.05) in at least one prenatal period. Pair-wise comparisons of prenatal periods in each breed showed that only the *CSRP3, LEF1, MRAS* and *MYOG* genes had higher expression (p < 0.05) in at least one prenatal period in commercial and Piau pigs. Overall, these results identified the *LEF1* gene as a primary candidate to account for differences in muscle mass between the pig breeds since activation of this gene may lead to greater myoblast fusion in the commercial breed compared to Piau pigs. Such fusion could explain the different muscularity between breeds in the postnatal periods.

## Introduction

Myogenesis is a prenatal process that involves the formation of muscle fibers through changes in gene expression and cell phenotype, and is influenced by genetic and environmental factors (Te Pas *et al.*, 2005). The size and number of muscle fibers determine muscle mass since skeletal muscle growth depends on the number of fibers formed during myogenesis and on postnatal muscle hypertrophy, which is limited by physiological and genetic factors (Rehfeldt *et al.*, 2000).

During prenatal development, two waves of myoblast proliferation and fusion give rise to primary and secondary muscle fibers (Wigmore and Evans, 2002). Primary muscle fibers are formed *de novo* in the early stages of myoblast fusion (first wave of differentiation) and secondary fibers use the primary fibers as a template in a second wave of differentiation (Rehfeldt *et al.*, 2000; Te Pas *et al.*, 2005); the latter fibers account for the majority of fibers in skeletal muscle (Beermann *et al.*, 1978). The number and size of primary myotubes are intrinsic factors that affect the number of secondary fibers. The number of secondary myotubes is sensitive to external factors such as nutrition, while the number of primary myotubes is genetically programmed and is unaffected by exogenous influences (Maltin *et al.*, 2001). In pigs, the waves of muscle fiber formation involve relatively long periods of time, i.e., ~30-60 days and 54-90 days of gestation for the first and second waves, respectively (Wigmore and Stickland, 1983).

The changes in gene expression associated with muscle development and growth have been examined in different breeds of pigs at various prenatal ages (Te Pas *et al.*, 2005; Cagnazzo *et al.*, 2006; Murani *et al.*, 2007; Sollero *et al.*, 2011; Zhao *et al.*, 2011). The analysis of changes in myogenic gene expression during the prenatal period, when the two waves of myoblast fusion occur, can be very important for understanding the biochemical differences that contribute to distinct interbreed variations in the degree of muscularity and meat quality.

In this study, we analysed the expression of myogenes in a commercial pig line (the three-way Duroc, Landrace and Large-White cross) and Piau pigs, which differ in muscularity from the commercial breed, in order to assess possible differences in gene expression during myogenesis.

## Material and Methods

### Biological material

Embryos and fetuses were obtained by cesarean section from three unrelated pregnant gilts for each genetic group of pigs (local Piau and commercial breeds) at 21, 40, 70 and 90 days post-insemination (dpi) maintained at the Pig Breeding Farm of the Departamento de Zootecnia at the Universidade Federal de Viçosa (UFV). Embryos and fetuses collected from the commercial breed were obtained from gilts of the three-way Duroc, Landrace and Large-White cross. Samples from three unrelated fetuses and embryos were used as biological replicates in gene expression analysis for each breed at 21, 40, 70 and 90 dpi. The procedures for obtaining the embryos and fetuses were approved by the Ethics Committee for Animal Use at UFV (protocol no. CEUA-UFV 85/2013), in accordance with current Brazilian federal legislation.

*Longissimus dorsi* (LD) muscle was collected at all periods, except from 21 dpi embryos, for which the whole individual was collected and used in RNA extraction. Samples were immediately placed in Falconer tubes containing 10 ml of RNA holder solution (BioAgency Laboratórios, Brazil) and sent to the Animal Biotechnology Laboratory at the Departamento de Zootecnia (UFV) where they were stored overnight at 4 °C and then transferred to a freezer at −80 °C until RNA extraction.

### RNA extraction

Total RNA was extracted using TRIzol^®^ reagent (Life Technologies, USA). The RNA was quantified in a NanoVue Plus spectrophotometer (GE Healthcare, Germany) and RNA A_260_/A_280_ ratios of 1.8-2.0 were used as an indicator of purity. The quality and intactness of extracted RNA were verified by electrophoresis in a 1% agarose gel. Total RNA was stored at −70 °C until cDNA synthesis.

### cDNA synthesis

Prior to the reverse transcription step, RNA was treated with DNase I amplification grade (Invitrogen, USA) to remove contaminant DNA, according to the manufacturer's instructions. The first strand of complementary DNA (cDNA) was synthesized using a GoScript^TM^ reverse transcription system (Promega, USA), according to the manufacturer's recommendations. cDNA concentrations were estimated spectrophotometrically in NanoVue^TM^ plus (GE Healthcare) and single stranded cDNA was stored at −20 °C until used in qPCR assays.

### Gene selection and primer design for qRT-PCR

The genetic data for differentially expressed genes (DEG) from an RNAseq experiment were used to compare gene expression between breeds in embryos and fetuses of a commercial pig breed (a two-way Landrace and Large-White cross) and Piau pigs (unpublished data). HUGO Gene Nomenclature Committee (HGNC) symbols for genes were obtained with the BIOMART/ENSEMBL program using ENSEMBL transcript identifications for DEG in conjunction with the pig (*Sus scrofa*) database. When HGNC symbols were not available for pigs, they were obtained by orthology using the *Homo sapiens* database. The HGNC symbols for genes were subsequently subjected to gene ontology analysis.

Information on gene ontology for the genes was obtained using the ToppCluster program (Kaimal *et al.*, 2010). The terms related to muscle development (myogenesis) were identified and their genes was used in metabolic pathway analysis. Metabolic pathway maps from KEGG (Kyoto Encyclopedia of Genes and Genomes) were obtained using DAVID software (Dennis *et al.*, 2003; Huang *et al.*, 2009). Cytoscape software (Shannon *et al.*, 2003) was used to view and edit the biological processes, molecular functions and metabolic pathways identified with ToppCluster (Kaimal *et al.*, 2010) and DAVID (Dennis Jr. *et al.*, 2003; Huang *et al.*, 2009) software. In addition, gene functions were evaluated using a Gene Cards database (Safran *et al.*, 2010) that provided concise information on all known and predicted human genes, in addition to information on gene ontologies that was not obtained by Toppcluster (Kaimal *et al.*, 2010).

Based on these findings, 13 genes were selected for analysis of their expression profiles using RT-qPCR. This work focused on genes that were differentially expressed between breeds because they represented primary candidates for information on the source of variation in muscularity and meat quality. The selected genes were related to myogenesis and were chosen based on gene ontology, the identification of metabolic pathways and their function.

qPCR primers were designed using PrimerQuest^®^ software (Owczarzy *et al.*, 2008) and nucleotide sequences obtained from the *S. scrofa* transcriptome database at GenBank (Benson *et al.*, 2013). The only nucleotide sequence not available for pigs was that of RSPO3, for which a homologous sequence from humans (*Homo sapiens*) was used. Table 1 summarizes relevant information for the genes that were studied, including the accession numbers of the transcript sequences used in primer design, the nucleotide sequences of the primers and amplicon size.

**Table 1:** GenBank accession numbers, primer sequences and amplicon sizes of the genes analyzed in this study.

Gene	Accession number	Primer sequences (5’ → 3’)	Amplicon size^1^
CHD8	XM_003482263.1	F: AGTGAGGACGAGAAGGAAGA	104
		R: GGGAATCCATCTTGGGACATAG	
CSRP3	NM_001172368.1	F: CAGCAACCCTTCCAAGTTCA	91
		R: CATCACCTTCTCAGCAGCATAG	
EID2B	XM_003127131.1	F: CGCCACTATCTGGAACACTAC	122
		R: CGCTGATATTCGGCATCAAAC	
HIF1AN	XM_003359328.1	F: GTACTGGTGGCATCACATAGAG	118
		R: CTGATGGGCTTTGAGAGGATATT	
IKBKB	NM_001099935.1	F: GATGGCGACAGTCAGGAAAT	107
		R: TTGCAAACCACCGTCTTACT	
LEF1	NM_001129967.1	F: CTATTGTAACGCCTCAGGTCAA	99
		R: TTGGCTCTTGCTCCTTTCTC	
MAP2K1	NM_001143716.1	F: GGAGCTGGAGCTGATGTTT	110
		R: GTCGGCTGTCCATTCCATAA	
MRAS	XM_003358570.2	F: GGTCGATTTGATGCATTTGAGG	96
		R: TCCTTGGCACTGGTTTCTATG	
MYOG	NM_001012406.1	F: CAGGCTCAAGAAGGTGAATGA	118
		R: GCACTCGATGTACTGGATGG	
RBM24	XM_001925447.3	F: TACCTGCCCACTATGTCTATCC	118
		R: GCAGCTCCCGTGTAATCAAT	
RSPO3	NM_032784.4	F: GAAACACGGGTCCGAGAAATA	110
		R: CCCTTCTGACACTTCTTCCTTT	
SOX7	XM_003359052.1	F: TCTCCACTCCAACCTCCA	120
		R: TCATTGCGATCCATGTCCTC	
SUFU	XM_001928912.4	F: GGAGCCCTCATTCCTCTTTG	83
		R: GCCATGTCACCTGTGATACTT	
ACTB^2^	XM_003124280.3	F: AAGATCAAGATCATCGCGCCTCCA	108
		R: ACTCCTGCTTGCTGATCCACATCT	
GAPDH^2^	NM_001206359.1	F: ACAGTCTTCTGGGTGGCAGTGAT	176
		R: CATGTTTGTGATGGGCGTGAACAA	
HPRT1^2^	NM_001032376.2	F: GCTGACCTGCTGGATTACAT	101
		R: CTGGTCATTACAGTAGCTCTTCAG	

1 Amplicon size in nucleotide number,

2 Reference gene. CHD8 – chromodomain helicase DNA binding protein 8, CSRP3 – cysteine and glycine-rich protein 3, EID2B – EP300 interacting inhibitor of differentiation 2B, HIF1AN – hypoxia inducible factor 1, α subunit inhibitor, IKBKB - inhibitor of κ light polypeptide gene enhancer in B-cells, kinase β, LEF1 – lymphoid enhancer-binding factor 1, MAP2K1 – mitogen-activated protein kinase kinase 1, MRAS – muscle RAS oncogene homolog, MYOG – myogenin (myogenic factor 4), RBM24 – RNA binding motif protein 24, RSPO3 – R-spondin 3, SOX7 – SRY (sex determining region Y)-box 7, SUFU – suppressor of fused homolog, ACTB – β-actin, GAPDH – glyceraldehyde-3-phosphate dehydrogenase, HPRT1 – hypoxanthine phosphoribosyltransferase 1.

### Testing of the designed primers

Primer amplification was assessed with the polymerase chain reaction (PCR) using 75 ng of pooled cDNA derived from embryo and fetal tissues of commercial and Piau pigs. Primers were tested at 200 nM and an annealing temperature of 60 °C in a Veriti^®^ 96-well thermal cycler (Applied Biosystems, USA). PCR was done with a GoTaq^®^ Green master mix kit (Promega) according to the manufacturer's protocol. The amplification products were screened for reaction specificity and the presence of primer dimers by electrophoresis on 8% polyacrylamide gels at 100 V for 2 h.

### Real time qPCR

Real time qPCR reactions were run in an ABI Prism 7300 Sequence Detection Systems thermocycler (Applied Biosystems) using a Gotaq^®^ qPCR master mix kit (Promega) according to the manufacturer's protocol. cDNA (25, 75 or 225 ng) or nucleic acid-free water (negative control) was added to each well of the plate along with upstream and downstream primers at 100, 200 or 400 nM.

The amplification conditions were: 95 °C for 2 min, 40 denaturation cycles at 95 °C for 15 s, and annealing and extension at 60 °C or 61 °C for 60 s. The efficiency of amplification was assessed after 40 amplification cycles by including an additional step in which the temperature was gradually raised from 60 °C to 94 °C to obtain the primer dissociation curve.

Target and reference gene amplifications were done in different wells of the same plate. The assays were run with three biological and two technical replicates for each treatment in a single 96-well plate. The coefficient of variation, used as an indicator of precision and reproducibility, was less than 5%, which was adequate for the Ct (threshold cycle) values of the technical replicates within each sample.

### Amplification efficiency

To calculate the amplification efficiency of target and reference genes, all reactions were done in 96-well plates using pooled cDNAs containing biological replicates of treatments and two technical replicates for each treatment. From the real time qPCR raw data, the Ct values and log_10_ amount of cDNA (25, 75 and 225 ng) were plotted in graphs for primers at 100, 200 and 400 nM. The slope of the resulting relationship was determined by linear regression and was used to calculate the amplification efficiency with the following equation, modified from Rasmussen (2001), in which efficiencies equal to 1 represent 100%:

$$E=10^{\left(\frac{-1}{\text{slope}}\right)}-1$$

Based on the results obtained in the preceding step, the highest amplification efficiency of target and reference genes was chosen, along with the appropriate primer concentration and quantity of cDNA for each gene to be used in the final qPCR reactions. Amplification efficiencies of 0.80 to 1.0 were considered appropriate (Table S1). The suitability of three reference genes (ACTB, GAPDH and HPRT1) for qRT-PCR was investigated using NormFinder software (Andersen *et al.*, 2004), and GAPDH was selected for data normalization since the gene used for this procedure should show no change in expression along the treatments. Ct values for a specific gene were normalized to the Ct value of highest expression (Ct minimum value) for that gene. The normalized Ct values were then used in the following equation (McCulloch *et al.*, 2012):

$$Q=E^{\left(\text{min Ct}–\text{Ct sample}\right)},$$

where: Q = normalized Ct value for a gene in the current sample, E = calculated amplification efficiency (ranging from 1 to 2, in which 100% = 2), min Ct = minimum Ct value for a gene among all samples, and Ct sample = Ct value for current sample and gene.

### Statistical analysis

The experimental design was a completely randomized 2 (breeds) x 4 (prenatal ages) factorial design with six repetitions (three biological and two technical replicates) per treatment. ANOVA was done using the following statistical model:

$$Y_{\text{ijkl}}=\text{μ}+A_{(\text{ij})I}+D_{\text{ijk}}+{\left(\text{RIG}\right)}_{\text{ijk}}+{\text{ε}}_{\text{ijkl}},\text{ where}$$

Y_ijkl_ is the expression level of gene k, in animal l, breed i and prenatal age j, in which i = 1 or 2 (commercial or Piau breed, respectively) and j = 1, 2, 3 or 4 (21, 40, 70 or 90 dpi, respectively),

μ is the general constant,

A_(ij)l_ is the random effect of animal l in breed i and age j,

A_(ij)l_ ~ N(0, σ^2^_A_),

D_ijk_ is the sample-specific random effect (common to both genes), D_ijk_ ~ N(0, σ^2^_D_),

(RIG)_ijk_ is the interaction effect between breed i and age j in gene k, and

ε_ijkl_ is the random error, i.e., ε_ijkl_ ~ N(0, σ^2^_e_).

This model was fitted to the data using the %QPCR_MIXED macro in SAS (Statistical Analysis System Institute Inc., USA), which is based on linear mixed models (Steibel *et al.*, 2009). The significance of contrast estimate values was assessed using Student's *t*-test. Contrast estimate values correspond to ΔΔCt and were used to assess relative expression (fold-change) by using the formula 2^–ΔΔCt^ (Livak and Schmittgen, 2001). In all cases, the level of significance was set at p < 0.05.

## Results

**Figure 1:**
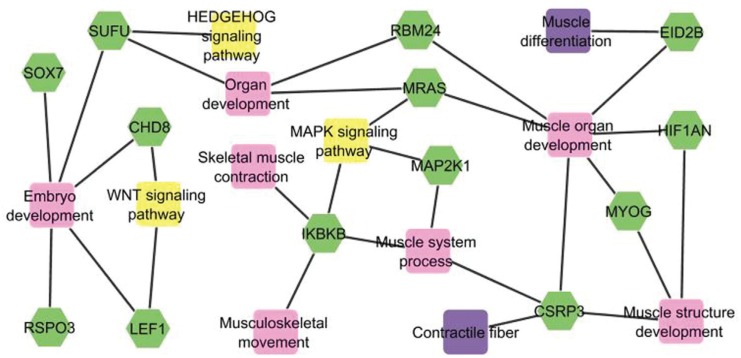
Functional gene networks and their interactions, showing the relationship between 13 genes (green). Twelve important subnets related to muscle development were included in biological process (pink), molecular function (violet) and metabolic pathway (yellow).

To understand the role of the 13 selected genes, information on gene ontology was obtained using Toppcluster software and metabolic pathways were investigated using DAVID software. These 13 genes are part of relevant functional metabolic networks for muscle development (Figure 1). These networks are for skeletal muscle contraction, muscle structure development, embryo development, organ development, muscle organ development, muscle differentiation, contractile fiber, musculoskeletal movement, muscle system process, and HEDGEHOG, MAPK and WNT signaling pathways. In addition, Table 2 describes the function of these 13 genes as defined in gene ontology terms.

**Table 2:** Metabolic pathways and gene ontologies for genes represented in the gene network.

		Gene ontologies		
Gene	Metabolic pathway	Cellular component	Molecular function	Biological process
CHD8	WNT signaling pathway	Nuclear lumen	Histone and DNA binding	Embryo development
CSRP3	–	Cytoskeleton	Contractile fiber	Muscle organ development/Muscle structure development/Muscle system process
EID2B	–	Nucleus	Muscle differentiation	Muscle organ development
HIF1AN	–	Nucleus/cytosol	Protein binding	Muscle organ development/Muscle structure development
IKBKB	MAPK signaling pathway	Nucleus/cytosol	Protein binding	Muscle system process/Musculoskeletal movement/Skeletal muscle contraction
LEF1	WNT signaling pathway	Nucleus	Chromatin and DNA binding	Embryo development
MAP2K1	MAPK signaling pathway	Cytoskeleton/cytosol/nucleus	Protein kinase activity	Muscle system process
MRAS	MAPK signaling pathway	Intracellular	GTPase activity/nucleotide binding	Muscle organ development/Organ development
MYOG	–	Nucleus	Chromatin and DNA binding	Muscle organ development/Muscle structure development
RBM24	–	Nucleus/cytoplasm	Nucleotide binding	Muscle organ development/Organ development
RSPO3	–	Extracellular region	Receptor binding	Embryo development
SOX7	–	Nucleus/cytoplasm	Nucleic acid binding	Embryo development
SUFU	Hedgehog signaling pathway	Nucleus/cytoplasm	Transcription corepressor activity	Embryo development/Organ development

**Figure 2:**
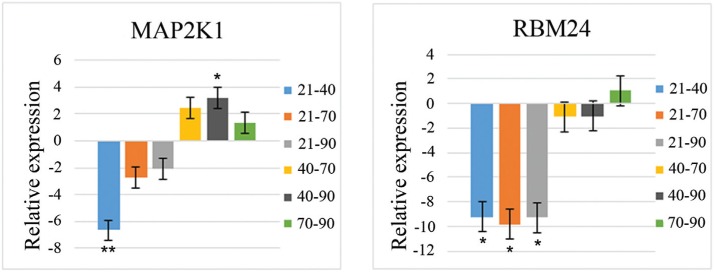
Relative expression levels for two genes (MAP2K1 and RMB24) in pair-wise comparisons of prenatal ages (21, 40, 70 and 90 days post-insemination). These genes differed significantly in relation to the factor ‘Period’ (p < 0.05, F-test in ANOVA), but showed no significant difference for the interaction ‘Breed x Period’. *p < 0.05 and **p < 0.01 indicates significant pair-wise comparisons by Student's *t*-test. A positive fold-change means that the first period in the comparison shows greater expression than the second period. Negative fold-change means that the second period in comparison presents greater expression than the first period.

qRT-PCR assays were done at four prenatal ages (21, 40, 70 and 90 dpi) in commercial and Piau pigs and the data were analyzed using ANOVA (Table 3). Seven genes (*CHD8, EID2B, HIF1AN, IKBKB, RSPO3, SOX7* and *SUFU*) showed no significant alterations, indicating that their expression was constant over time and between breeds. Two genes (*MAP2K1* and *RBM24*) showed significant changes (p < 0.05) in relation to the prenatal period, but there was no significant Breed x Period interaction for these genes. Table 4 shows the Student's *t*-test results for pair-wise comparisons among the prenatal periods for these genes. The relative gene expression (fold-change) for pair-wise comparisons of prenatal periods is shown in Figure 2. Of the genes analyzed, *MAP2K1* showed greater expression at 40 dpi (period of primary fiber formation) and 70 dpi (period of secondary fiber formation), whereas *RBM24* showed greater expression at 40 dpi (period of primary fiber formation) and at 70 and 90 dpi (periods of secondary fiber formation).

**Table 3:** P-values for ANOVA in relation to Breed, Period and interaction Breed x Period for the genes studied.

Genes	Factors		
	Breed	Period	Breed x Period
CHD8	0.9764	0.3615	0.6094
CSRP3	0.8615	**< 0.0001**	**< 0.0001**
EID2B	0.9072	0.1615	0.4284
HIF1AN	0.5757	0.2535	0.5533
IKBKB	0.7473	0.6656	0.6948
LEF1	0.9772	**< 0.0001**	**0.0004**
MAP2K1	0.4445	**0.0125**	0.0712
MRAS	0.6557	**0.0019**	**0.0205**
MYOG	0.7314	**< 0.0001**	**< 0.0001**
RBM24	0.9866	**0.0284**	0.1756
RSPO3	0.7198	0.0885	0.2230
SOX7	0.3451	0.4870	0.6915
SUFU	0.6504	0.2974	0.4175

Values in bold were statistically significant (p < 0.05) by F-Test;

**Table 4:** P-values for two-period comparisons for the genes MAP2K1 and RBM24. The ANOVA results (F-test) for these genes were significant for the factor Period.

Genes	Comparisons					
	21d x 40d^a^	21d x 70d	21d x 90d	40d x 70d	40d x 90d	70d x 90d
MAP2K1	**0.0027**	0.0772	0.1882	0.1177	**0.0456**	0.6143
RBM24	**0.0192**	**0.0165**	**0.0188**	0.9412	0.9917	0.9495

a 21d, 40d, 70d and 90d indicate the prenatal ages. Values in bold were statistically significant (p < 0.05).

**Figure 3:**
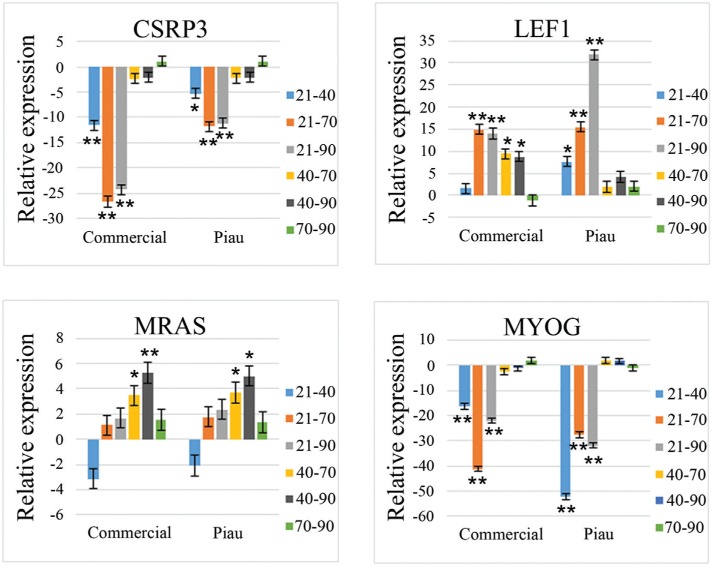
Relative expression for four genes (CSRP3, LEF1, MRAS and MYOG) in pair-wise comparisons of prenatal ages (21, 40, 70 and 90 days post-insemination) in commercial and Piau pigs. These genes showed a significant interaction for Breed x Period (p < 0.05, F-test in ANOVA). *p < 0.05 and **p < 0.01 indicates significant pair-wise comparisons by Student's *t*-test. A positive fold-change means that the first period in the comparison shows greater expression than the second period. Negative fold-change means that the second period in the comparison presents greater expression than the first period.

Four genes (*CSRP3, MRAS, LEF1* and *MYOG*) showed a significant (p < 0.05) Breed x Period interaction and Period factor based on ANOVA. Table 5 shows the Student's *t*-test results for comparisons that assessed a Breed x Period interaction for these genes. The relative gene expression (fold-change) for pair-wise comparisons of prenatal periods is shown in Figure 3 and revealed Breed x Period interactions (p < 0.05).

**Table 5:** P-values for two-period comparisons in commercial and Piau pigs. The ANOVA results (F-test) for these genes showed a significant Breed x Period interaction.

Genes	Breed	Comparisons					
		21d x 40d^a^	21d x 70d	21d x 90d	40d x 70d	40d x 90d	70d x 90d
CSRP3	Commercial	**0.0032**	**0.0003**	**0.0003**	0.2524	0.3070	0.8964
	Piau	**0.0309**	**0.0029**	**0.0035**	0.2719	0.3062	0.9365
LEF1	Commercial	0.5880	**0.0049**	**0.0058**	**0.0155**	**0.0183**	0.9356
	Piau	**0.0257**	**0.0045**	**0.0007**	0.4091	0.1052	0.3973
MRAS	Commercial	0.0575	0.8639	0.3663	**0.0412**	**0.0089**	0.4609
	Piau	0.1992	0.3261	0.1414	**0.0318**	**0.0107**	0.6007
MYOG	Commercial	**0.0026**	**0.0002**	**0.0012**	0.2565	0.7099	0.4366
	Piau	**0.0001**	**0.0006**	**0.0004**	0.4341	0.5396	0.8629

a 21d, 40d, 70d and 90d indicate the prenatal ages. Values in bold were statistically significant (p < 0.05) by Student's *t*-test.

Figure 3 and Table 5 show that commercial and Piau pigs had similar expression patterns for *CSRP3, MRAS* and *MYOG. CSRP3* showed greater expression during the two waves of myoblast fusion that gave rise to primary fibers at 40 dpi and secondary fibers at 70 and 90 dpi. *MRAS* showed greater expression during somite formation and proliferation at 21 dpi and during primary fiber formation at 40 dpi. *MYOG* had a greater expression at 40 dpi (period of primary fiber formation) and at 70 and 90 dpi (period of secondary fiber formation). *LEF1* showed a divergent expression pattern for commercial and Piau pigs: expression was greater at 21 dpi (period of somite formation and proliferation) and 40 dpi (period of primary fiber formation) in commercial pigs, whereas in Piau pigs this gene showed greater expression only at 21 dpi.

## Discussion

Thirteen genes related to important gene networks for muscle development and structure were analyzed by qRT-PCR to determine their expression profiles at 21 dpi (embryos) and at 40, 70 and 90 dpi (fetuses) in commercial (three-way Duroc, Landrace and Large-White cross) and local Piau pigs. In 21 dpi embryos we examined the region that would give rise to muscle, and which should contain only undifferentiated mesenchymal stem cells (also known as somite cells), since a histochemical study of pig embryos at 20 dpi found only somites in this region (Swatland and Cassens, 1973). In the other periods analyzed, primary myotubes were formed at 40 dpi and secondary myotubes developed at 70 and 90 dpi after myoblast differentiation. The analysis of myogene expression in these periods shed light on possible differences in myogenesis and subsequent muscularity in commercial and Piau pigs. Local Piau pigs are expected to have a higher level of fat than commercial pigs (Serao *et al.*, 2011), which have a greater muscle mass.

Muscle mass is influenced by the number and size of its muscle fibers (Rehfeldt *et al.*, 2000). We therefore focused on the expression of genes involved in the formation of myogenic cells in four periods since differential gene expression between genetically distinct lineages and periods could explain differences in muscle mass between breeds. Muscle development is a complex biological process regulated by various genes that interact with each other and a series of signal transduction pathways (Zhao *et al.*, 2011). Myogenic regulatory factors are controlled by regulatory pathways that activate or repress their activity, although additional factors are also probably involved in various transcription circuits that control myogenesis (Kong *et al.*, 1997).

*MAP2K1* showed similar expression in both breeds, with greater expression during primary and secondary fiber formation (40 and 70 dpi, respectively). This gene is involved in the MAPK signaling pathway that is important for muscle development since it can activate transcription factors involved in differentiation (Keren *et al.*, 2006), such as MEF2A, MEF2C and MYOD (Wu *et al.*, 2000) that can accelerate myoblast differentiation (Ulloa *et al.*, 2007). MAP2K1 can inhibit and activate myogenesis, depending on the developmental stage (Jo *et al.*, 2009), and can negatively control the switch from myoblast proliferation to differentiation by suppressing MYOD activity in the early stages of myogenesis (Perry *et al.*, 2001). In contrast, the presence of MAP2K1 protein in the nucleus of proliferating myoblasts can have a stimulatory role on muscle differentiation (Jo *et al.*, 2009). Thus, the differential expression at 40 dpi and 70 dpi suggests that MAP2K1 may have a stimulatory role in myoblast differentiation in myotubes of both breeds.

*RBM24* showed a similar expression pattern in commercial and Piau pigs, with greater expression during primary and secondary fiber formation (at 40 dpi and 70-90 dpi, respectively). *RBM24* is involved in biological processes related to muscle organ development. RBM24 protein interacts with *MYOG* mRNA to regulate the stability and expression of the latter through a post-transcriptional regulatory pathway (Jin *et al.*, 2010), but can also promote myogenic differentiation by modulating the cell cycle (Miyamoto *et al.*, 2009). The *RBM24* expression profile confirmed its role in controlling the stability and expression of *MYOG* mRNA and may therefore be involved in promoting primary and secondary fiber formation in both breeds. *MYOG* expression was also evaluated and showed a similar level of expression to *RBM24* throughout prenatal periods, as described below.

*MYOG* showed greater expression during the periods of primary (40 dpi) and secondary (70 dpi and 90 dpi) fiber formation in commercial and Piau pigs. This gene is related to the biological processes of muscle structure development and muscle organ development. MYOG is an important myogenic regulatory factor that is necessary for the formation of multinucleated myotubes (Keren *et al.*, 2006). These results confirmed the importance of this transcription factor for primary and secondary fiber formation during myogenic differentiation and showed that *MYOG* mRNA can be stabilized by RBM24 proteins since the synthesis of this mRNA is also high during primary and secondary fiber formation.

*CSRP3* showed greater expression during primary fiber (40 dpi) and secondary fiber (70 and 90 dpi) formation in commercial and Piau pigs. This gene is included in the molecular function of contractile fiber and in the biological processes of muscle system process, muscle organ development and muscle structure development. CSRP3 promotes myoblast differentiation and it is first expressed and accumulated in the nucleus when there is myotube formation and growth (Arber *et al.*, 1994). As shown here, *CSRP3* expression was enhanced during the two waves of myoblast differentiation in both breeds, in agreement with its role in primary and secondary fiber formation.

*LEF1* showed greater expression at 21 dpi and 40 dpi in commercial and at 21 dpi in Piau pigs, with lower expression thereafter. This gene belongs to the WNT signaling pathway and is related to the biological process of embryo development. The WNT pathway is important for muscle development because it can control the expression of myogenic regulatory factors such as MYF5 and MYOD, thereby influencing myogenic differentiation and survival (Cossu and Borello, 1999; von Maltzahn *et al.*, 2012). LEF1 can induce cellular cycle progression, cellular differentiation and apoptosis through transcriptional activation of *E2F1* (Zhou *et al.*, 2008). Based on the expression profile observed here, LEF1 is more important in the early stages of muscle development, mainly at 21 dpi when somites are formed and proliferate. The additional peak of expression seen at 40 dpi in commercial pigs indicates that LEF1 is possibly involved in the greater proliferation and fusion of myoblasts in this breed, which could account for the greater number of primary fibers in commercial pigs. Indeed, as mentioned above, LEF1 can induce cell cycle progression and cellular differentiation (Zhou *et al.*, 2008).

*MRAS* showed greater expression during the period of somite formation and proliferation (21 dpi) and primary fiber formation (40 dpi), with lower expression thereafter. This gene is related to the biological processes of muscle organ development and organ development and is involved in the MAPK signaling pathway. MRAS is a negative regulator of myoblast differentiation during myogenesis (Yokoyama *et al.*, 2007). Thus, we suggest that this gene has a greater role at 21 and 40 dpi, possibly by controlling somite formation and proliferation, as well as myoblast differentiation in primary fibers. MRAS can negatively regulate the expression and function of muscle-specific transcription factors such as *MYOD* and *MEF2* family (Lassar *et al.*, 1989; Winter and Arnold, 2000; Tortorella *et al.*, 2001) that are essential for controlling myoblast development and fusion to give rise to primary myotubes in both breeds. Myoblast differentiation into myotubes thus involves a balance between genes that activate and inhibit the process.

*CHD8, EID2B, HIF1AN, IKBKB, RSPO3, SOX7* and *SUFU* showed no changes in expression during the various periods or between breeds. Since there were no changes in gene expression, studies at the protein level are necessary in order to assess the roles of these genes in myogenesis in commercial and Piau pigs; differences in the amount of activated protein could be an important factor in distinguishing between the two breeds.

*CHD8, RSPO3, SOX7* and *SUFU* are related to embryo development. *CHD8* belongs to the WNT signaling pathway and can negatively regulate the transcriptional activity of various genes induced by activation of the WNT/β-catenin signaling pathway (Nishiyama *et al.*, 2012), in addition to preventing apoptosis (Nishiyama *et al.*, 2009). RSPO3 is a positive regulator of myogenesis in skeletal muscle by activating the WNT/β-catenin signaling pathway and can induce *MYF5* expression (Han *et al.*, 2011). SOX7 is a negative regulator of the WNT/β-catenin signaling pathway (Chan *et al.*, 2012) and can also be a tumor suppressor (Takash *et al.*, 2001). *SUFU* is related to the biological process of organ development and is included in the Hedgehog signaling pathway, of which it is a negative regulator by suppressing the activity and function of GLI transcription factors (Ding *et al.*, 1999). This suppression can increase the expression of specific muscle genes such as *MYOD* (Voronova *et al.*, 2013). The pathway is important for myogenesis because it is involved in cell proliferation and differentiation, tissue remodeling (Heretsch *et al.*, 2010), and specifies cellular growth and differentiation patterns (Rossi *et al.*, 2007).

*EID2B* and *HIF1AN* are involved in the biological process muscle organ development. *EID2B* is also related to muscle differentiation and can prevent myoblast differentiation into myotubes (Sasajima *et al.*, 2005). *HIF1AN* is also involved in the biological process of muscle structure development. This gene negatively regulates apoptosis (Yan *et al.*, 2011) and is important for myoblast differentiation, in which it acts as a crucial transcription factor that regulates myogenesis (Li *et al.*, 2007). Unaltered *HIF1AN* mRNA expression has already been observed during myogenesis (Wagatsuma *et al.*, 2011).

*IKBKB* is included in the MAPK signaling pathway and is related to the biological processes of skeletal muscle contraction, muscle system process and musculoskeletal movement. This gene can negatively regulate myoblast differentiation during myogenesis (Bakkar *et al.*, 2008).

Overall, the results of this study indicate that the *LEF1* gene is a primary candidate to explain the difference in muscularity between commercial and Piau pigs. Although this gene is expressed in both breeds, in commercial pigs this expression probably leads to greater fusion of myoblasts than in Piau pigs. In commercial pigs, *LEF1* showed an additional peak of greater expression at 40 dpi that corresponds to a critical period of myoblast proliferation and fusion during the first wave of myofiber formation. Thus, the greater number of fibers formed in commercial pigs compared to Piau pigs accounts for the greater muscularity seen in the former breed during postnatal development. The potential growth of skeletal muscle depends on the number of muscle fibers formed during the prenatal period and their postnatal hypertrophy (Rehfeldt *et al.*, 2000).

The findings reported here contribute to our understanding of the molecular mechanisms involved in muscle tissue formation in a commercial pig breed (the three-way Duroc, Landrace and Large-White cross) and Piau pigs. The new information on gene expression analyzed by qRT-PCR for the first time in pigs should be useful in understanding myogenesis and the possible mechanisms involved in the differences in muscularity between genetically distinct breeds.

## Supplementary material

The following online material is available for this article:

Table S1 - Amplification efficiency for target and reference genes.
