# Supplementary material for: Expression of myogenes in longissimus dorsi muscle during prenatal development in commercial and local Piau pigs
Source: Genet Mol Biol. 2016 Oct 31;39(4):589–99. doi: 10.1590/1678-4685-GMB-2015-0295 (PMC5127148; doi:10.1590/1678-4685-GMB-2015-0295)
Supplement: Supplementary file 1 [file 1415-4757-gmb-1678-4685-GMB-2015-0295-Suppl01.pdf]

Table S1 - Amplification efficiencies for target and reference genes.

	Gene	Temperature (°C)	Primer concentration (nM)	Quantity of cDNA (ng)	Amplification efficiency
Target genes	CHD8	61	100	25	0.95
	CSRP3	60	200	71	0.89
	EID2B	60	200	25	0.88
	HIF1AN	60	100	25	0.88
	IKBKB	61	200	25	0.99
	LEF1	60	200	75	0.99
	MAP2K1	60	200	75	0.99
	MRAS	60	200	25	0.81
	MYOG	61	100	25	0.93
	RBM24	61	100	25	0.99
	RSPO3	60	100	75	1.00
	SOX7	61	200	225	0.82
	SUFU	60	200	75	0.91
Reference genes	ACTB	61	100	75	0.93
	GAPDH	60	100	75	0.88
		61	100	75	0.95
	HPRT1	60	100	75	1.00
